# Harnessing the immune system: future directions in cancer immunotherapy

**DOI:** 10.3389/fimmu.2026.1777816

**Published:** 2026-04-16

**Authors:** Jingyao Zhang, Jiuwei Cui

**Affiliations:** Cancer Center, The First Hospital of Jilin University, Changchun, Jilin, China

**Keywords:** adoptive cell therapy, cancer immunotherapy, oncolytic viruses, tumor microenvironment, tumor vaccine

## Abstract

Cancer immunotherapy has remarkably reshaped the therapeutic landscape of oncology, and representative therapies including immune checkpoint inhibitors, adoptive cell therapy, oncolytic viruses and tumor vaccines have exhibited superior efficacy over conventional treatments in a broad spectrum of malignancies. Nonetheless, its clinical translation and application are still hampered by four core challenges: insufficient exploration of novel immune targets, inherent limitations of immunotherapeutic monotherapies, the lack of effective strategies for remodeling the microenvironment of cold tumors, and the immaturity of precise patient stratification systems. To address these aforementioned challenges, this review systematically outlines four core future development directions of cancer immunotherapy. First, the multi-dimensional excavation of novel therapeutic targets based on differential cellular expression molecules, metabolic regulatory networks, the tumor microenvironment and immune cell interactions. Second, the innovative upgrading of core therapeutic technologies encompassing engineered bacteria, oncolytic viruses, adoptive cell therapy and tumor vaccines. Third, the remodeling of the immunosuppressive microenvironment of cold tumors through the optimization of combination therapeutic strategies. Fourth, the construction of an integrated artificial intelligence-multi-omics model for precise patient stratification, so as to achieve dynamic prediction of therapeutic efficacy and accurate screening of populations that can derive the maximal benefit from immunotherapies. In the future, it will be necessary to deeply decode the dynamic interaction networks among tumors, the immune system, and the host. By integrating AI and multi-omics technologies, we should propel the evolution of cancer immunotherapy toward a closed-loop system featuring diagnosis, intervention and real-time monitoring, ultimately maximizing the clinical benefits of individualized treatment for cancer patients.

## 1 Introduction

Cancer immunotherapy has remarkably reshaped the therapeutic landscape of oncology and broken the inherent paradigm of conventional cancer treatments centered on the direct elimination of tumor cells. Its core mechanism lies in enabling the body to acquire an autonomous capacity for eradicating malignant tumors by activating anti-tumor immune responses or remodeling the functions of immune effector cells. Innovative therapeutic modalities represented by immune checkpoint inhibitors (ICIs), adoptive cell therapy (ACT), oncolytic viruses (OVs) and tumor vaccines have exhibited significantly superior efficacy to conventional chemoradiotherapy and targeted therapy in a variety of solid and hematologic malignancies (1, 2).

Although numerous studies have validated the “curative” potential of immunotherapy for cancer (3, 4), its widespread application still faces four core challenges: (1) Insufficient exploration of novel and effective immune targets. Current targets fail to fundamentally address issues including low treatment response rates (5), frequent emergence of drug resistance (6)and immune-related adverse events (irAEs) (7), which have become core bottlenecks limiting treatment efficacy; (2) Although ICIs, ACT, OVs and tumor vaccines constitute the four pillars of cancer immunotherapy, each therapy has inherent limitations—such as severe drug resistance to ICIs, insufficient targeting ability and complex preparation of ACT, suboptimal efficacy of OVs as monotherapies, and weak immunogenicity of tumor vaccines. Consequently, there is currently no ideal treatment regimen that combines high efficacy, high safety, and high accessibility; (3) A lack of systematic strategies for remodeling the tumor microenvironment (TME) of “cold tumors”. How to disrupt the immunosuppressive microenvironment, promote immune cell infiltration, and convert “cold tumors” into “hot tumors” that are sensitive to immunotherapy remains an urgent and critical unsolved problem (8); (4) The precision treatment system remains underdeveloped, with a shortage of efficient patient stratification methods and treatment decision-making models. This hinders the realization of personalized treatment tailored to individual patients, leading some patients to receive ineffective therapy or suffer from unnecessary adverse reactions.

Based on the above four core challenges, the future development of cancer immunotherapy will focus on four targeted dimensions as follows: (1) Elucidation of novel immune regulatory mechanisms and development of therapeutic targets, which focuses on overcoming the barriers to tumor immune recognition, fundamentally improving treatment response rates, and alleviating drug resistance and irAEs; (2) Technological innovation-driven upgrading of therapeutic modalities, compensating for the inherent deficiencies of the four conventional core therapies through technological advancement, and enhancing the implementation efficacy and clinical accessibility of immunotherapy; (3) Optimization of combination therapy strategies, achieving a synergistic effect of “1 + 1>2” through in-depth exploration of rational mechanistic combinations, effectively remodeling the TME and addressing the therapeutic dilemma of “cold tumors”; (4) Construction of precise population identification models, relying on multi-omics, artificial intelligence (AI) and other technologies to realize accurate patient stratification and personalized treatment decision-making, so as to maximize therapeutic benefits and minimize treatment risks.

**Figure 1:**
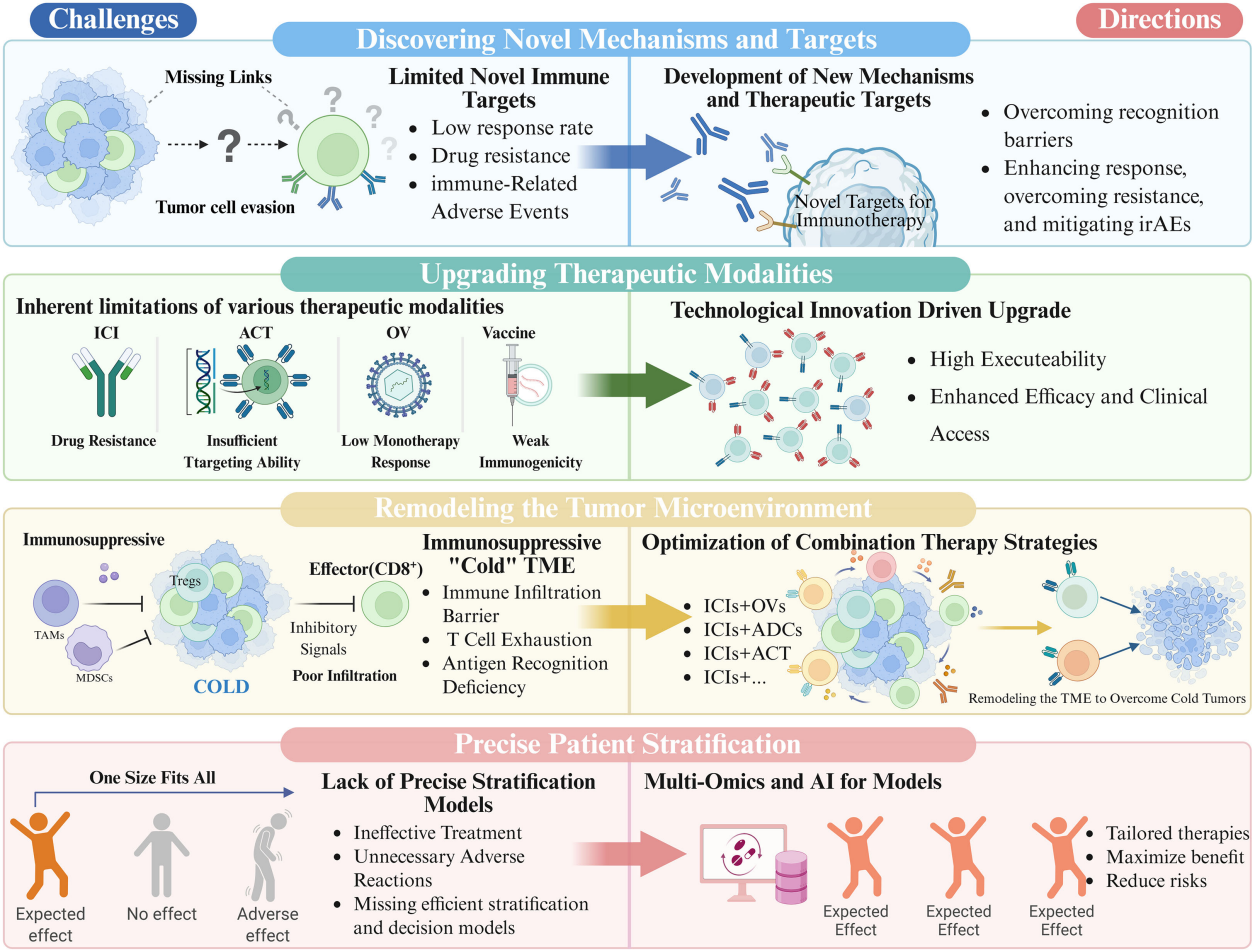
The core challenges and corresponding future developmental directions in cancer immunotherapy. Created in https://BioRender.com.

Centering on the above core framework, this review systematically summarizes the research progress of cancer immunotherapy, conducts an in-depth analysis of the critical challenges currently faced, and prospects its future development directions, thereby providing theoretical support and practical references for the basic research and clinical translation of next-generation cancer immunotherapy. (**Figure 1**).

## 2 Elucidation of novel immune regulatory mechanisms and target development

**Figure 2:**
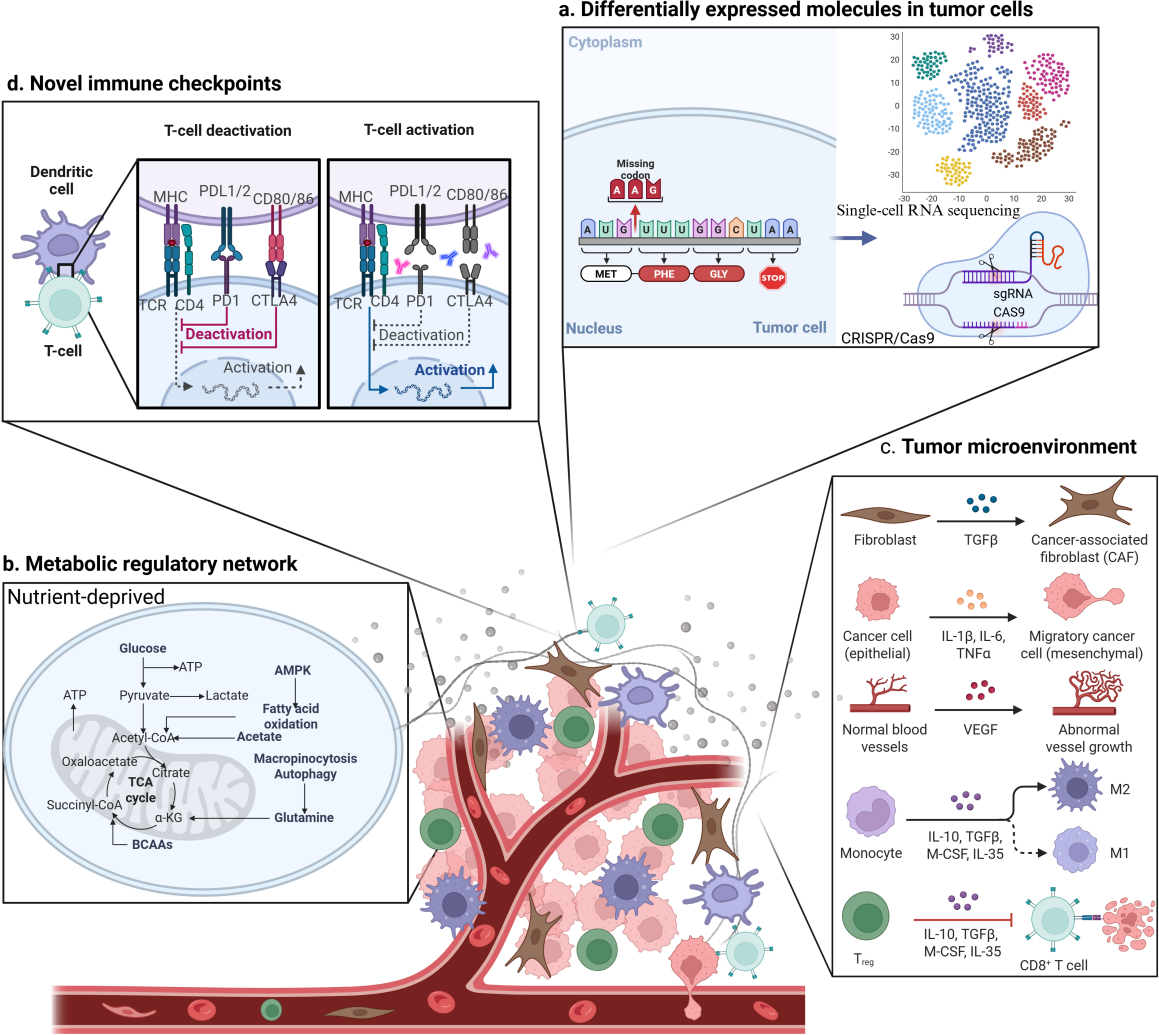
Identify novel targets for tumor immunotherapy by screening from the perspectives of **(a)** differentially expressed molecules in tumor cells, **(b)** metabolic regulatory networks, **(c)** the tumor microenvironment (TME), and **(d)** novel immune checkpoints. Created in https://BioRender.com.

The efficacy of cancer immunotherapy hinges on deep analysis and precise intervention of immune regulatory networks. In recent years, research has explored four key dimensions: differentially expressed molecules in cells, metabolic regulatory networks, heterogeneity of the TME, and novel immune checkpoints. (**Figure 2**).

### 2.1 Identifying novel therapeutic targets by investigating the impact of differentially expressed molecules in tumor cells on anti-tumor immune responses

Research on the regulatory mechanisms of anti-tumor immune responses mediated by differentially expressed molecules in tumor cells has identified multiple novel targets for tumor immunotherapy.

Tumor cells construct intricate immune evasion networks through differentially expressed molecules. With breakthroughs in technologies such as CRISPR-Cas9 screening and RNA sequencing (RNA-seq), screening strategies based on differentially expressed genes have uncovered numerous innovative targets for tumor immunotherapy (9). For instance, recent studies indicate that differentially expressed molecules in tumor cells—such as TMBIM1 (10), SMARCAL1 (11), CD28 (12), and ZBTB46 (13), which are differentially expressed in tumor cells, all promote the formation of an immunosuppressive TME and thus impair the efficacy of immunotherapy. Beyond tumor cells, screening of other components within the TME has also revealed novel immune regulatory molecules associated with cancer immunotherapy (14–17), providing new targets and therapeutic directions for cancer immunotherapy.

Despite these breakthroughs, the aforementioned technologies exhibit limitations such as incomplete genomic coverage and oversimplified cellular states, leading to the omission of potential therapeutic targets. A recent innovative dual-pathway CRISPR screening technology has overcome this bottleneck: by independently performing CRISPR-based loss-of-function screens in naive CD8^+^ T cells and activated CD8^+^ T cells, researchers identified the E3 ubiquitin ligase STUB1 and its adaptor protein CHIC2 as a novel negative regulatory complex for the antitumor function of CD8^+^ T cells (18). Furthermore, given the difficulty in establishing causal relationships between gene perturbations and immune-related phenotypes, researchers developed the Immune-related CRISPR screen analyzer of functional targets (ICRAFT), which integrates immune-related CRISPR screening datasets, single-cell RNA sequencing (scRNA-seq) data, and pre-treatment RNA sequencing data from clinical trials. ICRAFT successfully identified multiple dual-effect targets that both suppress tumor progression and enhance anti-tumor immune responses (19).

However, while genomic screening remains a primary method for uncovering gene functions, it performs poorly in identifying secreted proteins. Recent clinical studies have identified potential immunotherapy targets by integrating clinical omics data. The Cancer Immunology Data Engine (CIDE) platform, constructed from 90 multi-omics datasets spanning 17 solid tumor types and 5,957 patients, successfully identified the secreted protein acyloxyacyl hydrolase (AOAH) as a regulator of tumor immunity. AOAH specifically clears immunosupressive arachidonoyl phosphatidylcholines and oxidized derivatives, thereby reversing tumor immune suppression (20).

These findings elucidate the multifaceted molecular strategies employed by tumors to evade immune surveillance, while providing precise intervention directions to reverse immunosuppression. In the future, systematic analysis of tumor cell differential expression profiles through integration of multi-omics data will become a critical pathway for discovering novel immunotherapeutic targets.

### 2.2 Identifying novel metabolic intervention targets through the perspective of metabolic regulatory network analysis

Metabolic reprogramming, a hallmark of cancer, not only serves as a critical underpinning for tumors to adapt to the microenvironment and achieve malignant proliferation, but also acts as a pivotal “metabolic checkpoint” that modulates the efficacy of cancer immunotherapy (21), thus emerging as a cutting-edge and highly focused research direction in the field of tumor immunology. The metabolic reprogramming of tumor cells creates a severe conflict with the metabolic demands of immune cells. The crosstalk between their metabolic pathways determines the response rate and durability of immunotherapy directly through three core mechanisms: nutrient competition, accumulation of suppressive metabolites, and metabolic remodeling of immune cells, which constitutes an important intrinsic cause mediating tumor immune evasion and immunotherapy resistance.

On the one hand, tumor cells compete with immune cells for essential nutrients in the TME via metabolic reprogramming, directly triggering starvation-induced exhaustion of immune cells and thus serving as a major contributor to primary resistance to immunotherapy (22). In addition to the competition for glucose between tumor cells and CD8^+^ T cells under the classic Warburg effect (23), recent studies have revealed that tumor cells also consume key nutrients such as taurine (24) and methionine (25) in a competitive manner. This leads to metabolic deprivation in immune cells, which rely on nutrient substrates for activation and proliferation, causing them to enter a state of functional arrest and ultimately inducing irreversible exhaustion of T cells. Even if the signal inhibition of immune checkpoints is relieved by ICIs, the cytolytic function of T cells cannot be restored, ultimately resulting in therapeutic failure.

On the other hand, genetic or epigenetic mutations in key enzymes within the tumor metabolic regulatory network trigger aberrant accumulation of metabolites in the TME. As potent immunosuppressive signaling molecules, these metabolites construct a robust immunosuppressive barrier by directly inhibiting immune cell function and recruiting immunosuppressive immune cells (26, 27). For example, aberrant activation of the ATP-adenosine pathway represses *de novo* pyrimidine synthesis in T cells, which significantly impairs the anti-tumor capacity of T cells and becomes a major hurdle for the clinical efficacy of ICIs in solid tumors (28). Excessive lipids in the TME (29–31) also induce lipid peroxidation and accelerated exhaustion of effector T cells, compromising the long-term survival benefits of immunotherapy. Notably, it is well established that lactate accumulation reduces the pH of the TME, thereby inhibiting the function of CD8^+^ T cells while promoting the infiltration of regulatory T cells (Tregs) and myeloid-derived suppressor cells (MDSCs). However, recent studies using a sequential CRISPR screening strategy have identified that lactate acidification is a core driver of tumor metabolic adaptation *in vivo*, which regulates mitochondrial fusion and promotes oxidative metabolism through the extracellular signal-regulated kinase - dynamin-related protein 1 (ERK-DRP1) pathway. This finding indicates that lactate is not merely a metabolic byproduct but also an important signaling molecule that actively shapes the metabolic network of cancer cells (32), providing a novel direction for the treatment of acidic TME; the combination of mitochondrial fusion inhibitors with immunotherapy may yield superior therapeutic efficacy.

Although most current studies have been conducted from the perspective of the metabolic crosstalk interface between tumor and immune cells, aiming to screen dual-functional targets that can both specifically inhibit the malignant proliferation of tumor cells and abrogate metabolic immunosuppression to enhance the anti-tumor function of immune cells. For instance, screening using metabolic compound (33) libraries and genome-wide CRISPR-Cas9 revealed that dihydroorotate dehydrogenase (DHODH) drives tumor cell macropinocytosis, increasing intracellular lysine and tryptophan uptake, thereby suppressing major histocompatibility complex (MHC)-II molecule expression in tumor cells. Inhibition of DHODH expression in tumor cells effectively recruits more immune cell infiltration and provokes anti-tumor immune responses *in vivo*, thus overcoming resistance to Programmed Death-1 (PD-1) inhibitors (33). The mitochondrial outer membrane channel protein voltage-dependent anion channel 2 (VDAC2) inhibits BAK activation, blocking the mitochondrial apoptosis pathway and forming an immunosuppressive barrier. Targeting VDAC2 in tumor cells promotes interferon-γ (IFN-γ)-induced cell death and activates the cGAS-STING pathway, converting “cold” tumors into immunologically “hot” tumors with immune cell infiltration, significantly enhancing antitumor efficacy and immune therapy response (34). However, these targets remain in the preclinical or early clinical stages, with suboptimal real-world therapeutic efficacy. A potential underlying reason is that tumor cells and immune cells often share homologous metabolic enzymes. Therefore, there is an urgent need to develop selective inhibitors that can distinguish between homologous metabolic enzymes in tumor cells and immune cells, so as to avoid simultaneously inhibiting the metabolic activity of immune cells and thus attenuating anti-tumor immune responses (35).

In addition, metabolic targets exhibit distinct cell subset specificity in their regulatory effects, with the same metabolic target exerting diametrically opposed functions in different immune cells or cell subsets. For example, activation of pyruvate kinase M2 (PKM2) enhances the effector functions of CD8^+^ T cells through metabolic reprogramming and mitochondrial remodeling, whereas PKM2 agonists inhibit the activation, proliferation and cytokine production of CD4^+^ T cells (36). This imposes higher demands on the precise development of metabolic targets for cancer immunotherapy.

### 2.3 Identification of novel immune regulatory cell intervention targets from the perspective of the TME

The TME, as the core arena for dynamic interactions between immune cells and tumor cells, has seen its immune cell heterogeneity and plasticity deeply elucidated. In recent years, it has emerged as a hotspot for discovering novel immune regulatory cells and intervention targets (37).

Traditional TME research has focused on T lymphocytes, B lymphocytes, tumor-associated macrophages (TAMs), neutrophils, and MDSCs (38). However, recent studies have revealed additional functional cell subpopulations. A groundbreaking study identified a CD4^+^ T cell subset with stem-like properties, which exhibits both self-renewal potential and plasticity to differentiate into classical CD4^+^ effector T cells. Its differentiation trajectory is strictly regulated by Tregs: in the immunosuppressive microenvironment mediated by Tregs, these stem-like CD4^+^ T cells are confined to a low-differentiated state and primarily differentiate toward induced Tregs (iTregs), thereby significantly suppressing the antitumor activity of effector CD8^+^ T cells. When Tregs are specifically depleted, these stem-like CD4^+^ T cells undergo phenotypic remodeling to differentiate into T helper 1 (TH1) cells, which drive CD8^+^ T cell effector differentiation via IFN-γ secretion. Further mechanistic studies confirmed that forced expression of the transcription factor TBET can break through the immunosuppressive barrier established by Tregs and restore the antitumor efficacy of CD4^+^ T cells (39). Through combined scRNA-seq and epigenomic sequencing analysis, a study in hepatocellular carcinoma (HCC) identified two cancer-associated fibroblast (CAF) subsets—CAF-FAP and CAF-C7—with opposing prognostic implications. CAF-FAP colocalizes with PD-1^+^ CD8^+^ T cells, and in an orthotopic HCC model, combined inhibition of CAF-FAP and anti-PD-1 therapy induces more effective tumor regression than either monotherapy (40). Multi-omics analysis revealed that PLAUR^+^ neutrophils in HCC drive immunosuppression by TAMs and CD8^+^ T cell exclusion, serving as a key regulator of immunotherapy resistance, and targeting PLAUR^+^ neutrophils offers a new direction for HCC immunotherapy (41). B cells exhibit complex functional heterogeneity in tumor immunity, and precise subpopulation classification holds significant therapeutic value for immunotherapy. Tumors can induce two distinct patterns of systemic B cell abnormalities: Tumor-induced B cell abnormality type 1 (TiBA-1) and Tumor-induced B cell abnormality type 2 (TiBA-2). TiBA is closely associated with the prognosis of triple-negative breast cancer (TNBC) patients receiving neoadjuvant immunotherapy, with TiBA-1 and TiBA-2 patients showing significantly lower rates of complete pathological response compared to TiBA-0 patients (tumors that do not induce B cell abnormalities) (42). Additionally, studies showed that plasma cells have two distinct differentiation pathways: the germinal center (GC) and extra-follicular (EF) pathways. Cancers dominated by the EF pathway (e.g., HCC) are associated with immune dysregulation and worse clinical outcomes (43).

Classic immune cells also exhibit non-traditional functions within the TME. For example, mast cells, once considered pro-tumor “accomplices”, have recently been shown to enhance the efficacy of programmed cell death-ligand 1 (PD-L1) inhibitors, suggesting that targeting mast cells may represent a novel strategy to potentiate immunotherapy (44). TAMs can suppress anti-tumor immunity by cultivating Tregs. Specifically, Arg1-expressing TAMs secrete the chemokine platelet factor 4 (PF4) in the TME, promoting Treg polarization toward TH1-Tregs and thereby weakening the immune response. Specific depletion of arginase 1 (Arg1)^+^ TAMs or neutralization of PF4 inhibits tumor growth and enhances antitumor immunity (45).

As a multidimensional hub of the immune regulatory network, the TME continues to unveil underappreciated immune cell subsets and regulatory axes through multidimensional technological innovations, paving new avenues to overcome current therapeutic bottlenecks.

### 2.4 Identification of novel immune regulatory cell intervention targets from the perspective of new immune checkpoints

Immune checkpoint molecules play a critical role in maintaining immune homeostasis by regulating the activation and exhaustion of immune cells, while tumor cells often hijack these pathways to achieve immune evasion. ICIs,—specifically inhibitors targeting PD-1/PD-L1 and cytotoxic T-lymphocyte-associated protein 4 (CTLA-4)—have now been approved as standard treatments for over 15 cancer types (2, 46). Landmark 10-year follow-up data from the CheckMate-067 clinical trial demonstrated that nivolumab combined with ipilimumab not only significantly extended overall survival (OS) and disease-specific survival in melanoma patients but also exhibited favorable safety profiles, further validating the enduring survival benefits of immunotherapy (4). Nevertheless, most patients develop primary or acquired resistance, and a subset of patients experience irAEs, which has driven the exploration and clinical translation of novel immune checkpoint targets.

Although conventional novel ICIs have achieved certain clinical outcomes, multiple clinical trials including RELATIVITY-047 (47) and TACTI-002 (48), have validated that lymphocyte-activation gene 3 (LAG3) is a highly promising target for cancer immunotherapy. Furthermore, LAG3-targeted inhibitors were successfully approved in 2022, officially joining the ranks of standard clinical treatments. However, LAG3 inhibitors have encountered successive setbacks in a series of subsequent large-scale clinical trials. The Phase III RELATIVITY-098 study showed that Opdualag, a fixed-dose combination of Nivolumab and Relatlimab, failed to reach the primary endpoint of relapse-free survival (RFS) for the adjuvant treatment of patients with completely resected Stage III-IV melanoma (49). Similarly, LAG3 inhibitors have also shown unsatisfactory efficacy in the first-line treatment of non-small cell lung cancer (NSCLC), and the much-anticipated Phase III TACTI-004 clinical trial was recently terminated as it failed to meet the primary endpoints of progression-free survival (PFS) and OS (50). In addition, the clinical translation of other conventional novel immune checkpoint targets has also progressed sluggishly: clinical trials of inhibitors targeting T cell immunoreceptor with Ig and ITIM domains (TIGIT), such as SKYSCRAPER-02 (51) and KEYVIBE-008 (52), all failed to achieve the prespecified endpoints; multiple Phase III clinical trials of antibodies targeting T cell immunoglobulin and mucin domain 3 (TIM-3), including STIMULUS-MDS2 (53) and COSTAR Lung (54), also ended in failure.

Despite these successive setbacks, clinical research on targets such as LAG3, TIM-3 and TIGIT is still ongoing, and the emergence of bispecific antibodies (bsAbs) has brought hope for breaking this impasse. The ARTEMIDE-01 study indicated that Rilvegostomig, a PD-1/TIGIT bispecific antibody, exhibited encouraging efficacy and safety in patients with metastatic non-small cell lung cancer (mNSCLC) who had not received prior ICI treatment (55).

In addition, with the development of technologies such as CRISPR and scRNA-seq, a large number of novel immune checkpoints have been successively identified in preclinical studies. For example, studies have found that signaling lymphocytic activation molecule 6 (SLAMF6), an immune inhibitory receptor on T cells, suppresses T cell activation and anti-tumor immunity through cis-homophilic interaction; its high-potency blocking monoclonal antibody can significantly enhance the activation of human and murine T cells, reduce the exhausted phenotype, and inhibit tumor growth (56). ITPRIPL1, the first naturally occurring ligand for the CD3 complex, transmits inhibitory signals by directly binding to the CD3ϵ subunit. Currently, clinical trials for ITPRIPL1 antibody therapy against tumors have successfully obtained Investigational New Drug (IND) approval from the U.S. Food and Drug Administration (FDA) and are in Phase I clinical studies (57).

Despite the current challenges such as immune resistance and patient selection bias, with the in-depth advancement of research, novel ICIs are expected to further enrich the pipeline of cancer immunotherapy, improve the survival prognosis of patients, and bring more possibilities for clinical benefits to cancer patients.

## 3 Technological innovation-driven evolution of therapeutic paradigms

(**Figure 3**).

**Figure 3:**
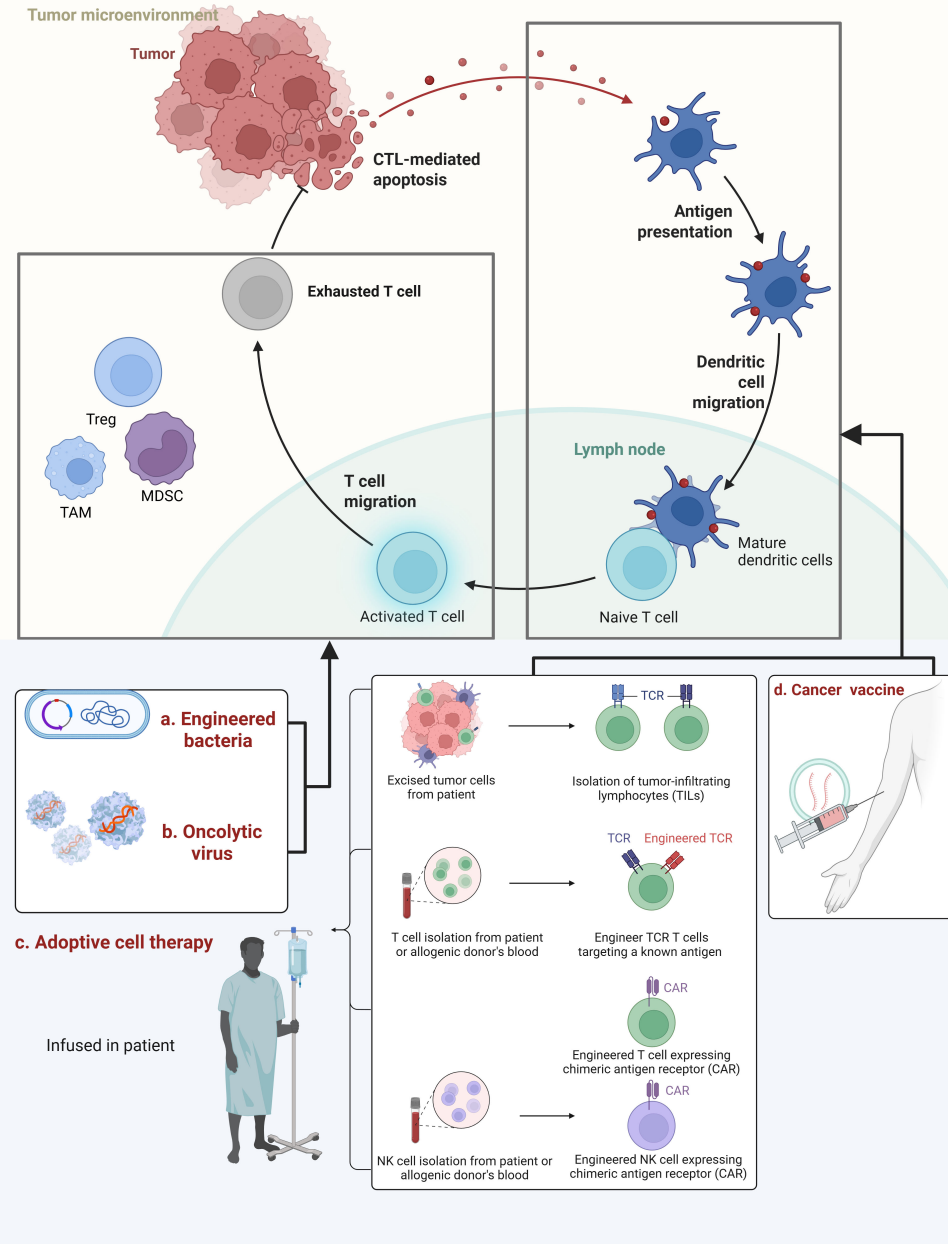
The TME hosts a dynamic interplay between immune activation and suppression. During normal antitumor immune activation, dendritic cells (DCs) migrate to capture tumor antigens, mature within the TME, and migrate to lymph nodes. There, they present antigens to naive T cells, activating them into activated T cells. These activated T cells migrate to the TME, where they should eliminate tumors via cytotoxic T cell killing. However, they become ineffective due to “exhaustion” caused by the suppressive TME. Technologies such as engineered bacteria, OVs, ACT, and tumor vaccines can intervene at different stages of the immune process to break suppression, enhance T cell activation, and ultimately achieve antitumor therapy. **(a)** Engineered bacteria are genetically modified to target tumor tissues while stimulating the host immune response, thereby disrupting tumor immune suppression. **(b)** OVs: Utilizing genetically engineered viruses to specifically infect and lyse tumor cells, releasing tumor antigens to further activate the immune system and enhance antitumor efficacy. **(c)** ACT involves isolating immune cells (e.g., tumor-infiltrating lymphocytes, T cells, NK cells) from the patient or donor. These cells undergo *in vitro* genetic engineering (e.g., TCR or CAR introduction) to precisely recognize tumor antigens. After expansion, they are reinfused into the patient to achieve targeted tumor killing. **(d)** Tumor vaccines deliver tumor antigens to the immune system through vaccination, activating specific T-cell immune responses. This proactively induces the body to develop anti-tumor immune memory, preventing tumor recurrence or suppressing tumor growth. Created in https://BioRender.com.

### 3.1 Engineered bacteria

Engineered bacteria, leveraging their immune-stimulatory properties and preferential proliferation in hypoxic, immunosuppressive TME, have emerged as a promising novel antitumor therapeutic strategy (58). Their core advantage lies in achieving TME-specific activation through synthetic biology modifications. Optimizing bacterial functions by integrating gene editing with surface functionalization techniques can significantly enhance therapeutic efficacy while reducing adverse effects (59, 60).

As innovative drug delivery vehicles, engineered bacteria require precisely controllable drug expression-release regulation systems to prevent potential toxicity from non-specific expression in normal tissues. Current research, through multiple modification strategies, has endowed engineered bacteria with multidimensional regulatory capabilities to respond to bacterial quorum-sensing signals, exogenous physical/chemical stimuli, or TME-specific markers. For instance, genetically engineered bacteria encoding the co-stimulatory molecule ligand OX40 ligand (OX40L) have been conjugated with a novel sonosensitizer, enabling spatiotemporally controllable separation via ultrasound irradiation (61). Another study developed the HRB@LM composite system. After specifically targeting tumor tissues, liposomes (LMs) respond to locally elevated glutathione concentrations to rapidly release macrophage colony-stimulating factor (M-CSF), triggering macrophage tumor infiltration. This process induces anti-CD47 antibody synthesis via hypoxia-induced TME, enhancing macrophage-mediated tumor cell killing (62). The near-infrared (NIR) light-mediated PadC-based photoswitch (NETMAP) system confers near-infrared light responsiveness to engineered bacteria, enabling precise spatiotemporal release of therapeutic payloads in the TME (63). Genetic engineering of the probiotic *Escherichia coli* Nissle 1917 enables specific expression of adenosine deaminase in tumor hypoxic environments, converting immunosuppressive adenosine into immunostimulatory inosine to remodel the TME (64). A heat shock-inducible IL-15/IL-15Rα expression system constructed using attenuated *Salmonella typhimurium* VNP20009 colonizes tumor sites after intravenous injection, with cytokine expression triggered by microwave ablation (MWA), inducing antitumor immune responses that effectively inhibit tumor growth even with incomplete MWA (65).

Although engineered bacteria have brought new breakthroughs to bottlenecks in traditional tumor therapies, this novel technology still faces challenges in clinical translation. Safety is the primary prerequisite: while engineered bacteria exhibit targeting properties, as living therapeutic agents, their targeting efficacy is unstable, and systemic administration may cause severe reactions. Thus, balancing tumor-killing efficacy while reducing bacterial virulence is necessary to enhance targeting without compromising antitumor effects. Additionally, the efficacy of genetically engineered bacteria expressing immunotherapeutic drugs varies among patients, and expression levels significantly impact safety. Despite precise regulation achieved via synthetic biology, these issues remain key bottlenecks hindering clinical translation and require further exploration.

### 3.2 OVs

**Figure 4:**
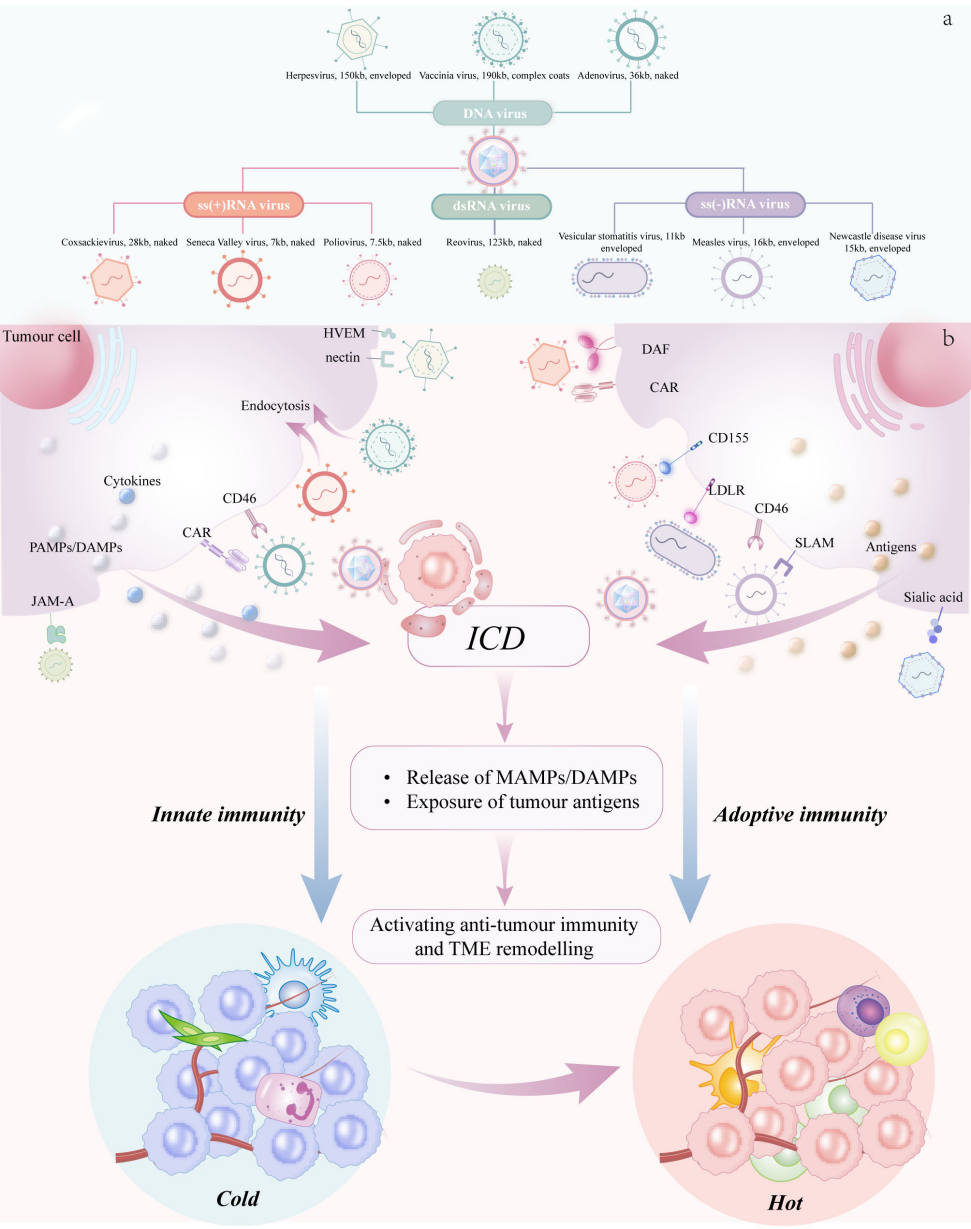
Classification and Mechanisms of OVs. **(a)** shows the classification of OVs. **(b)** illustrates the mechanism of action of OVs. OVs exert dual antitumor effects by selectively infecting and replicating within malignant cells, triggering lytic cell death. This process releases tumor-specific antigens alongside pathogen-associated molecular patterns (PAMPs) and damage-associated molecular patterns (DAMPs)—molecules that serve as immune-stimulatory signals. These activate DCs and other antigen-presenting cells (APCs), bridging innate and adaptive immunity. The resulting immune response targets both infected and uninfected tumor cells through the cytotoxic activity of antigen-specific T cells.

OVs, as a new generation of cancer treatment strategy, overcome the limitations of traditional therapies through the synergistic action of multiple mechanisms, including direct lysis of tumor cells, activation of systemic anti-tumor immune responses, and disruption of the tumor vasculature. They particularly offer innovative solutions for converting “cold” tumors into “hot” tumors (66, 67). Based on the type of viral nucleic acid, OVs are classified into two major categories: RNA viruses and DNA viruses. DNA viruses have more mature clinical research due to their large genomic size that facilitates genetic engineering, whereas RNA viruses possess unique advantages in that they do not integrate into the host cell genome and their small genome enables efficient crossing of the blood-brain barrier (68). (**Figure 4**).

In 2015, based on positive results from the OPTIM clinical trial (69), T-VEC was approved by the US FDA for the treatment of melanoma (70), becoming the world’s first marketed OV drug and marking a milestone breakthrough in this field. With the advancement of gene editing and synthetic biology technologies, OVs have experienced a second wave of development following the era of tumor immunotherapy ushered in by ICIs. The academic community generally anticipates that OVs can break through core bottlenecks such as insufficient delivery efficiency, restricted administration routes, undefined beneficiary populations and limited monotherapeutic efficacy. However, in reality, a decade has passed since T-VEC’s approval, and no second OV product has been marketed worldwide. This phenomenon stems not from a single technical bottleneck, but from flawed logic across the entire pipeline from preclinical research to clinical translation in this field.

Specifically, Current genetic engineering strategies for OVs primarily focus on four areas: deleting viral pathogenicity genes, inserting immunomodulatory factors, modifying receptor targeting, and enhancing antiviral evasion. Newcastle disease virus with porcine α1,3GT gene (NDV-GT) generates the xenogenic antigen αGal on tumor surfaces to trigger hyperacute immune rejection, demonstrating a 50% survival extension in preclinical liver cancer models and a 90% disease control rate (DCR) with no severe toxicity in 23 treatment-resistant advanced cancer patients (71); however, the clinical benefits of this strategy have only been validated in small, single-arm studies. The reproducibility of results in large-scale randomized controlled trials and the risk of additive toxicity when combined with ICIs remain unclear, which is a common bottleneck faced by the vast majority of genetically modified OVs. VG161, a genetically engineered herpesvirus that deletes the neurotoxicity gene ICP34.5 while carrying IL-12, IL-15/IL-15Rα, and PD-1/PD-L1 blocking proteins, has demonstrated significant activity in advanced hepatocellular carcinoma (72) and sarcoma (73), making it one of the candidates currently progressing most rapidly in translation; However, it should be noted that while the co-expression of multiple cytokines can enhance immune activation, it also carries the potential risk of a cytokine storm, and the safety window for clinical dosing still requires further clarification through large-scale studies.

Beyond genetic modification, delivery efficiency constitutes another core determinant of the clinical efficacy of OVs. An ideal delivery system should achieve specific enrichment of viruses at tumor sites, while enabling evasion of clearance by the host immune system and minimization of off-target toxicity to normal tissues (74). In current clinical applications, intratumoral injection remains the primary administration route for OVs (75–77). Although this route achieves high local viral enrichment at tumor sites, it is only applicable to superficial, easily accessible tumors such as melanoma, and has extremely poor applicability for deep-seated tumors and multiple metastatic lesions, which is entirely unable to meet the clinical treatment needs of patients with advanced solid tumors. Therefore, extensive research has been conducted on systemic delivery strategies for OVs in this field, focusing on physical/chemical delivery approaches and biological carriers. For instance, the EV71@AM nanocapsule encapsulating recombinant OV EV-A71-miR124T demonstrates blood-brain barrier penetration for glioma treatment, offering a novel central nervous system tumor strategy (78). The oncolytic virus-T cell chimera (ONCOTECH) technology utilizes T cells to deliver CRISPR/Cas9-expressing OVs to tumors, enhancing targeted delivery to solid tumors while simultaneously improving the tumor microenvironment and boosting the efficacy of both OVs therapy and ACT (79). The RadioOnco formulation (AD@PSSP), modified with polyethylenimine-selenium-polyethylene glycol, improves intravenous delivery efficiency by protecting viruses from rapid clearance (80). However, most systemic delivery strategies have only been validated for feasibility in murine models, without consideration of key issues pertaining to clinical translation such as viral clearance in the human circulation, immunogenicity and the feasibility of large-scale manufacturing. Systemic delivery OVs that have truly entered the clinical stage remain scarce, and the delivery bottleneck remains the greatest obstacle restricting the development of OVs from localized therapy to systemic anti-tumor treatment.

Despite the enormous application potential demonstrated by OVs, their clinical value has been the subject of extensive debate compared with mature immunotherapeutic strategies such as ICIs, and numerous core bottlenecks in this field urgently need to be addressed. Real-world study data of T-VEC also suggest that OVs may only be suitable for patients with predominantly injectable lesions, relatively intact immune function and low disease burden (81), and their general applicability awaits further validation.

### 3.3 ACT

ACT has emerged as a cutting-edge technology in tumor immunotherapy, with variations primarily stemming from the source of immune cells and engineering strategies (82). Chimeric antigen receptor-T cells (CAR-T) and T cell receptor-engineered T cells (TCR-T) confer precise tumor-targeting capabilities and sustained efficacy to peripheral blood-derived T cells via genetic engineering, demonstrating advantages in hematologic malignancies and achieving breakthroughs in certain solid tumors (83–87). Tumor-infiltrating lymphocyte (TIL) therapy, by expanding and activating T cells extracted from tumor tissues, leverages their rich tumor-specific TCR repertoire, natural tumor-homing properties, and robust tissue-infiltration capacity to achieve landmark progress in solid tumor treatment (88–90). Despite their respective strengths, these differences also create distinct therapeutic bottlenecks, contributing to the disconnect between preclinical/clinical research and clinical practice, with few FDA-approved solid tumor treatments.

Off-target toxicity remains a major obstacle limiting CAR-T cell therapy. Current strategies include optimizing single-chain variable fragment (scFv) affinity for novel CAR-T construction (91), incorporating logic gates (92) or inducible suicide genes (93), and screening new targets. Taking Satri-cel (a Claudin18.2-targeted CAR-T product) as an example, a Phase I clinical trial in CLDN18.2-positive advanced gastrointestinal cancer patients reported an objective response rate (ORR) of 38.8% and a DCR of 91.8%, with median progression-free survival (mPFS) and median overall survival (mOS) of 4.4 months and 8.8 months, respectively. Although 96.9% of patients experienced cytokine release syndrome, all cases were Grade 1-2, showing improved safety. Subgroup analysis revealed that patients without liver/bone metastases and high CLDN18.2 expression achieved 8.4-month mPFS and 13.1-month mOS (83). These results highlight precision target screening as a key efficacy driver while underscoring the need for combination strategies to overcome single-target limitations in solid tumors. Notably, combining CAR-T’s cytolytic power with TCR’s precise neoantigen recognition enhances specificity and reduces healthy tissue toxicity. This novel TCR/CAR-T cell therapy demonstrated superior anticancer activity and minimal off-target effects in preclinical/clinical studies, providing theoretical and experimental foundations for safer, more effective solid tumor CAR-T therapies (94).

Beyond off-target toxicity, CAR-T efficacy in solid tumors is constrained by target heterogeneity, immunosuppressive TME, T cell persistence, and infiltration challenges (95, 96). Promising engineering directions include: cytokine/immunomodulator “arming” (e.g., IL-10 (97), IL-15 (98), IL-12 (99), TGF-β pathway inhibition (100)) to enhance TME adaptation; multi-target CAR-T (101–103) or alternative cell types (e.g., CAR-NK (104, 105), CAR-γδ T (106)) to overcome heterogeneity; local delivery (107), stromal remodeling (108), and chemokine incorporation (109) to improve infiltration; and gene editing to enhance persistence and prevent exhaustion in TME (110). While primarily used in hematologic cancers, these approaches show solid tumor potential. Autologous CAR-T’s high costs, long production times, and limited scalability have driven universal CAR-T (UCAR-T) (111) and *in vivo* CAR-T manufacturing (112) advancements, marking a shift toward standardized, scalable applications.

TCR-T therapy, with broader antigen recognition and lower activation thresholds, faces similar challenges plus human leukocyte antigen (HLA) restriction limiting patient eligibility (113). Solutions require multidimensional research: TCR affinity tuning, neoantigen discovery, manufacturing optimization, and combination strategies to advance safer, more efficient clinical applications.

TIL therapy circumvents CAR-T/TCR-T bottlenecks by selecting tumor-reactive natural T cells, with the first FDA-accelerated approval of Lifileucel for PD-1/PD-L1-refractory melanoma (114). However, individual variability in tumor-reactive T cell frequency and IL-2-related toxicity remain key application barriers (115, 116). Gene editing offers innovative solutions to optimize TIL proliferation, cytokine profiles, cytotoxicity, persistence, and reduce IL-2 dependency (117–121).

Notably, despite FDA accelerated approval, Lifileucel’s safety profile shows 87.7% Grade 4 treatment-related adverse events, 23.6% ICU admission rate, and 7.5% treatment-related mortality, necessitating Phase III trials to confirm clinical value (114). Additionally, complex manufacturing and specialized regulatory requirements limit widespread adoption, particularly in developing countries. Future TIL improvements must prioritize safety optimization, process simplification, and regulatory framework enhancement.

### 3.4 Tumor vaccine

**Figure 5:**
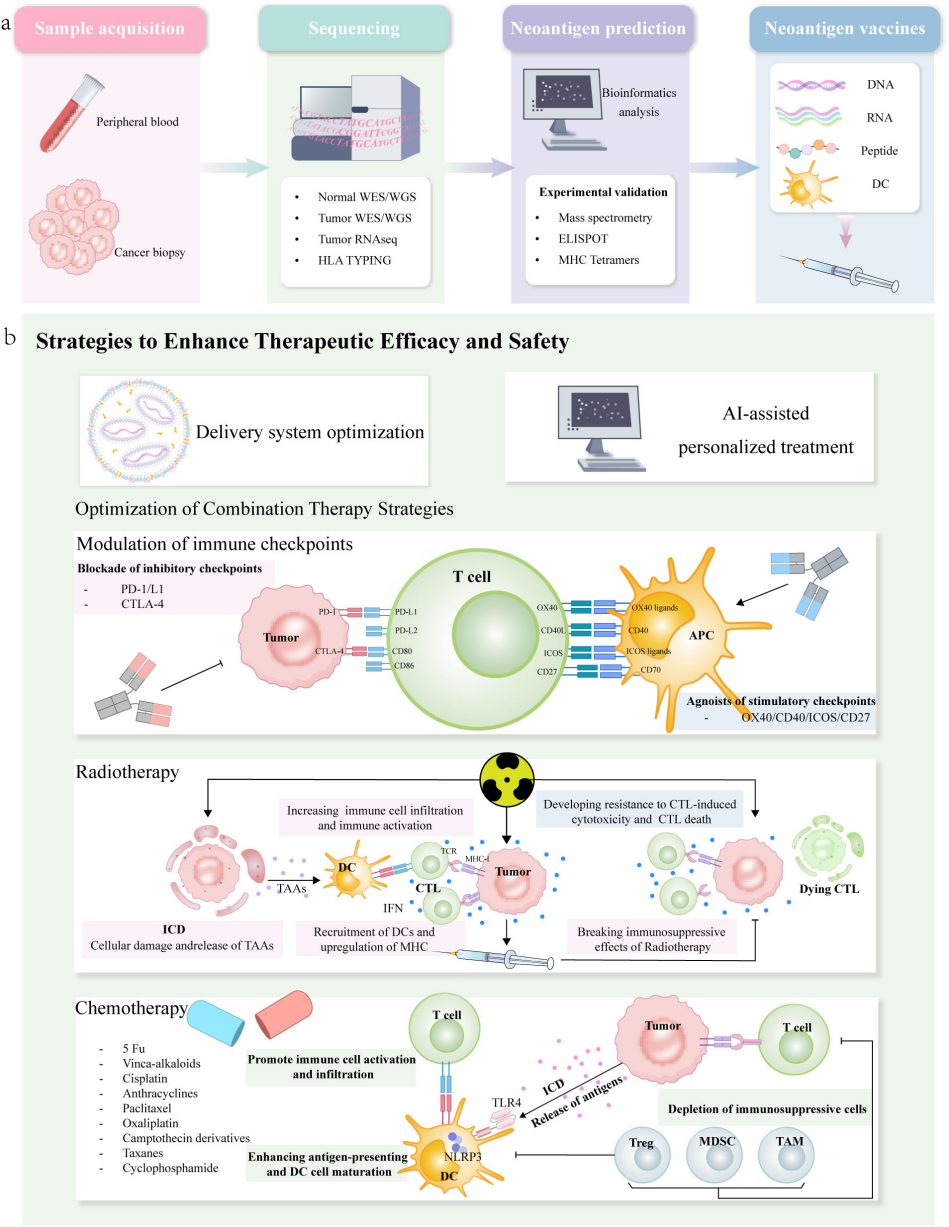
**(a)** Preparation process of personalized neoantigen tumor vaccines and strategies to enhance their therapeutic efficacy, includes the following steps: sample acquisition, sequencing analysis, neoantigen prediction and validation, as well as vaccine preparation and administration. However, the efficacy of tumor vaccines as monotherapy is limited. **(b)** depicts current combination therapy strategies that can enhance the efficacy of tumor vaccines involve integrating approaches such as immune checkpoint inhibitors, radiotherapy, and chemotherapy, which further amplify the antitumor immune response.

Tumor vaccines, as an innovative branch of immunotherapy, primarily target tumor-associated antigens (TAAs) and tumor-specific antigens (TSAs) to induce cytotoxic T lymphocytes and other immune effector cells to generate a durable immune response (122). The optimal breakthrough point for tumor vaccine development currently lies in selecting the most suitable antigens that can induce anti-tumor immune responses while avoiding anti-autoimmune reactions and autoimmune toxicity (123). Neoantigens, which are protein sequences generated by tumor mutations and genetic alterations and absent in normal cells, exhibit high tumor specificity and serve as ideal vaccine targets. In pancreatic cancer patients vaccinated with ELI-002, a shared neoantigen-targeted tumor vaccine, remarkable anti-tumor efficacy was observed, with a median OS of 28.94 months and a median recurrence-free survival (RFS) of 15.31 months (124). In recent years, the integration of omics technologies and AI algorithms has revolutionized the precision and efficiency of neoantigen prediction and personalized vaccine design (125, 126). (**Figure 5**).

The following are personalized neoantigen tumor vaccines currently in clinical trials across different platforms. (**Table 1**).

**Table 1:** Clinical Trials of Personalized Neoantigen Tumor Vaccines.

Platform	Vaccine	Target cancer	Route	Results	Phase/reference
peptide	iNeo-Vac-P01	22 patients with cancer	Subcutaneous	DCR was 71.4%	(127)
		with advanced stage		mPFS was 4.6 months	
		of various tumor types		mOS was not reached	
peptide	PGV001	10 patients with	Subcutaneous	2 participants treated	I (128)
		urothelial cancer		in the metastatic	
				setting achieved an OR	
peptide	NeoVax	9 patients with high-risk,	Subcutaneous	None of the 9	I (129)
		fully resected clear cell		participants had a	
		RCC(stage III or IV) with		recurrence of RCC	
		or without ipilimumab			
		administered adjacent			
		to the vaccine			
		8 patients with		All patients were	I (130)
		surgically resected stage		alive and six were	
		IIIB/C or IVM1a/b melanoma		without evidence of	
				active disease	
peptide	NeoVax^MI^	10 patients with previously	Subcutaneous	9 patients novel in	I (131)
		untreated melanoma		vitro T-cell responses	
		(stage IIIB/C/D, IVM1a–c)		were induced against	
				most of the	
				neoepitopes used for	
				immunization	
DNA	GNOS-PV02	Patients with advanced HCC	Intradermal	ORR was 30.6% and	Ib/IIa (132)
		previously treated with		8.3% of patients	
		a multityrosine kinase		achieved a CR	
		inhibitor			
DNA	Neoantigen	Patients with persistent	Electroporation	Recurrence-free	I (133)
	DNA vaccine	TNBC following neoadjuvant		survival was 87.5%	
		chemotherapy			
mRNA	BNT122	Patients with locally	Intravenous	One patient with	I (134)
		advanced or metastatic		gastric cancer	
		solid tumors		achieved a CR	
		Patients with PDAC	Intravenous	Responders with	I (135)
				vaccine-induced	
				T cells have	
				prolonged RFS	
mRNA and	GRANITE	Patients with colon cancer	Intramuscular	The median PFS was	II (136)
adenovirus		with minimal residual		12.88 months in the	
		disease following surgical		GRT-R902 combination	
		resection and standard		therapy group, compared	
		adjuvant chemotherapy		to 12.12 months	
				in the control group	
mRNA	mRNA-4157	Patients with completely	Intramuscular	RFS was longer with	II (137)
		resected melanoma		combination versus	
		(stage IIIB-IV)		monotherapy	
		Patients with resected	Intramuscular	Recruiting	I (138)
		NSCLC or			
		resected cutaneous melanoma			
DCs	Neo-DCVac	Patients with NSCLC	Subcutaneous	Recruiting	NCT06329908
			or lymph node		(139)

However, personalized neoantigen vaccines currently face numerous challenges, such as inefficient antigen prediction, incomplete patient data, and immune-suppressive TME that hinder T-cell infiltration and lead to T-cell exhaustion. Memorial Sloan Kettering Cancer Center reported in an mRNA personalized vaccine clinical trial that only 11% of bioinformatically predicted neoantigens were recognized by patients’ T cells (140). Current neoantigen prediction relies more on mutations, showing promising potential for tumors with high mutation burden; however, for tumor types with moderate or low mutation burden, there is a more urgent need for neoantigen epitopes independent of mutations.

Additionally, the choice of delivery systems and vaccine formulations is a critical determinant of tumor vaccine efficacy. Currently, lipid nanoparticle (LNP)-based delivery systems combined with mRNA vaccines have become the mainstream clinical delivery platform (137, 138, 141), and are the only FDA-approved delivery systems for human use (142). Multiple studies are currently focused on designing and optimizing LNPs to enhance tumor vaccine efficacy (143–146). While some Toll-like receptor agonists have been approved for prophylactic infectious disease vaccines, more robust data are needed to identify optimal combination strategies (125).

Future tumor vaccine development will require further optimization in areas such as neoantigen discovery, vaccine design refinement, and delivery system innovation.

## 4 Optimization of combination therapy strategies

ICIs, by blocking immunosuppressive signaling pathways such as PD-1/PD-L1 and CTLA-4 to remodel the host’s anti-tumor immune responses, have been established as one of the core therapeutic modalities in oncology. Nevertheless, monotherapeutic ICIs still face bottlenecks including limited response rates and the frequent development of drug resistance in most solid tumors. Combination therapy strategies centered on ICIs, which achieve synergistic modulation of the TME and potentiate immune activation effects, have emerged as a pivotal direction for overcoming suboptimal therapeutic efficacy. At present, these strategies primarily focus on combination therapies targeting the PD-1/PD-L1 axis, which are designed to augment therapeutic efficacy through diverse combinatorial approaches (147).

### 4.1 In combination with chemotherapy

Optimization strategies for ICIs combined with chemotherapy primarily focus on spatiotemporal sequencing, dosing kinetics, drug compatibility, and selection of treatment-responsive populations.

The selection of chemotherapeutic agents constitutes the fundamental determinant of therapeutic efficacy in combination therapy, as distinct chemotherapeutic drugs exhibit markedly divergent regulatory effects on the immune microenvironment, and not all agents can elicit a synergistic effect with ICIs. The IMpassion130 trial demonstrated that nab-paclitaxel combined with atezolizumab extended OS by 7.5 months in patients with advanced TNBC, earning FDA Breakthrough Therapy designation (148). In contrast, the highly similarly designed IMpassion131 trial, which adopted solvent-based paclitaxel plus atezolizumab, failed to observe significant survival benefits (149). Further mechanistic studies revealed that the combination of nab-paclitaxel and atezolizumab promotes T cell fate remodeling, B cell germinal center reconstitution, and myeloid cell functional reprogramming, significantly enhancing antigen presentation efficacy (44). This indicates that the combination of chemotherapy and ICIs is by no means a mere pharmacological combination; instead, the precise selection of chemotherapeutic agents based on their immunomodulatory properties is warranted, rather than the blind adoption of conventional chemotherapy regimens.

The sequence of chemotherapy and immunotherapy administration also impacts treatment outcomes. Preclinical studies in murine models of melanoma, lung metastases, and peritoneal metastases showed that “chemotherapy followed by immunotherapy” exhibited superior antitumor activity compared to “concurrent” or “immunotherapy followed by chemotherapy” (150). Sequential administration with chemotherapy first may circumvent chemotherapy’s suppression of CD8^+^ T cell differentiation from stem-like to terminally differentiated effector cells, preserving T cell expansion potential and IFN-γ secretion capacity, potentially representing a superior treatment strategy (151). A prospective study in patients with driver-negative advanced NSCLC corroborated this finding, identifying day 3 post-chemotherapy as the optimal timing for ICI administration, with significantly improved ORR, DCR, and mPFS compared to concurrent therapy (152). It should be noted, however, that a universal consensus has not yet been reached regarding the optimal administration time window and sequencing cycle for different tumor types and chemotherapy regimens, which remains a key unsolved issue for clinical translation in this field.

Conventional chemotherapy regimens based on the maximum tolerated dose are often accompanied by severe myelosuppression and systemic immune injury, which abrogate the synergistic effects with ICIs. In contrast, the optimization of dose regimens has ushered in a novel paradigm for balancing the efficacy and toxicity of combination therapy. Metronomic chemotherapy, through continuous low-dose administration, promotes vascular normalization and enhances immune cell infiltration (153), and when combined with ICIs, significantly improves PFS in metastatic breast cancer patients, particularly those with TNBC (154). Individualized dose adjustment also demonstrates translational potential, as the Neo-N study found that most patients with early-stage TNBC may achieve comparable efficacy with half the chemotherapy dose used in the KEYNOTE-522 regimen (155, 156), suggesting that biomarker-based dose stratification could optimize the risk-benefit ratio of immunotherapy combinations.

### 4.2 In combination with radiotherapy

Radiotherapy-ICI synergism has emerged as a research hotspot in oncology, though therapeutic strategies demonstrate significant heterogeneity in efficacy outcomes.

Clinical evidence highlights temporal modulation as a critical determinant of combination efficacy: the PACIFIC trial’s sequential consolidation ICI therapy after concurrent chemoradiotherapy established standard-of-care status in unresectable stage III NSCLC (157), whereas concurrent or early ICIs administration in PACIFIC2 failed to meet endpoints (158). Similarly, the positive outcome of KEYNOTE-A18 (159) and the failure of CALLA (160) further confirm the decisive impact of treatment sequence on the threshold for immune activation.

Radiotherapy exhibits a double-edged sword effect on the TME through multiple mechanisms: On one hand, significant therapeutic expansion of both clonal and pre-existing T cell clones is observed in tumors (distant sites) and blood (systemic) following radiotherapy combined with immunotherapy, accompanied by clonal neoantigen-reactive autologous T cell responses (161). Conversely, increased TGF-β secretion and MDSC infiltration may establish an immunosuppressive barrier (162). Dose fractionation optimization offers new perspectives to resolve this paradox—preclinical studies confirm that hypofractionated radiotherapy (HFRT) with single high-dose irradiation (≥5 Gy) triggers IFN-I responses (163), while low-dose radiotherapy (LDRT) reshapes immunosuppressive microenvironments via TAMs polarization (164). Both modalities demonstrate superior synergistic potential to conventional fractionation in clinical trials. LDRT combined with ICIs shows manageable safety and preliminary efficacy in extensive-stage small cell lung cancer (165), while HFRT-ICI combinations enhance tumor-specific T cell infiltration in solid tumors (166). Additionally, hybrid dose regimens combining high- and low-dose RT achieve balanced immune activation and suppression in animal models (167), with clinical translation under investigation in prospective trials (168).

Technological innovations have expanded the dimensions of radiotherapy-immunotherapy combinations: FLASH radiotherapy, through ultra-high dose rate delivery (>40Gy/s), preserves immune activation while significantly reducing normal tissue toxicity, demonstrating synergistic effects with ICIs (169). Spatially fractionated radiation therapy enables cascade activation of immune cells in and outside the irradiation field via sub-tumoral region-specific ablation (170). Boron neutron capture therapy using ^10^B/siPD-L1 nanoparticles establishes a novel paradigm for targeted radiotherapy-immunotherapy integration, achieving local tumor control while inducing systemic antitumor immune responses (171).

### 4.3 In combination with OVs

OVs can selectively lyse tumor cells, release tumor antigens and damage-associated molecular patterns (DAMPs), disrupt the immunosuppressive barrier of the TME, and convert “cold tumors” into “hot tumors”, thereby enhancing the sensitivity of tumors to ICIs. The combination of these two modalities exerts a synergistic anti-tumor effect (172, 173). In contrast to the limited efficacy of OVs as monotherapy, the combination with ICIs significantly improves the clinical response rate, furnishing a novel therapeutic option for patients with refractory tumors.

A Phase I clinical trial evaluating the efficacy of OVs as monotherapy and in combination with pembrolizumab in patients with advanced solid tumors demonstrated that ASP9801, an OV agent, was well-tolerated as a monotherapy but exhibited insufficient efficacy, whereas its combination with ICIs showed certain anti-tumor activity (174). The IGNYTE trial revealed that RP1 in combination with nivolumab yielded an ORR of 33.6% (47/140) in patients with advanced or metastatic melanoma who had failed or developed resistance to prior anti-PD-1 immunotherapy, with partial response (PR) and complete response (CR) rates of 18.6% and 15%, respectively. In terms of survival outcomes, the 1-year, 2-year and 3-year overall survival rates were 75.3%, 63.3% and 54.8%, with a relatively consistent response rate observed across all patient subgroups. This regimen thus provides a potential new standard of care for refractory patients who fail to benefit from conventional immunotherapy (175). VG161 combined with camrelizumab, a PD-1 inhibitor, exhibited remarkable anti-tumor activity in patients with advanced primary liver cancer, achieving an ORR of 18.2%, a mPFS of 6.3 months and a 6-month OS rate as high as 87.5% (176). CG0070 combined with nivolumab achieved pathological response rates comparable to cisplatin chemotherapy in patients with cisplatin-intolerant muscle-invasive bladder cancer (177). Neoadjuvant OV orienX010 plus toripalimab showed robust antitumor activity with high response rates and good tolerability in resectable acral melanoma (178). DNX-2401 combined with pembrolizumab demonstrated safe efficacy in GBM treatment, with significant survival prolongation in some patients (179). MEDI5395 (NDV-GM-CSF) combined with durvalumab effectively alleviated advanced solid tumors (180). TILT-123 combined with pembrolizumab showed excellent tolerability without dose-limiting toxicities in platinum-resistant/refractory ovarian cancer (181).

It should be noted, however, that most current studies on the OV-ICI combination are small-sample, early-phase clinical trials lacking Phase III confirmatory evidence. In addition, the systemic delivery efficiency, tumor targeting specificity and long-term safety of OVs remain major hurdles for their clinical translation.

### 4.4 In combination with ACT

ACT therapy directly kills tumor cells by activating or genetically modifying immune cells ex vivo, while ICIs restore T-cell function by blocking immunosuppressive pathways such as PD-1/PD-L1 and CTLA-4. Their combination exerts synergistic antitumor effects, offering a novel strategy for cancer treatment (182).

In metastatic breast cancer, a phase II trial confirmed significant efficacy of TILs combined with short-term pembrolizumab: patients treated with ACT plus neoantigen-specific TILs demonstrated objective tumor responses, with one patient maintaining CR for 5.5 years (183). Similar breakthroughs were observed in prostate cancer, where TIL-ICI combinations achieved pathological CR (184). In NSCLC treatment, the combination of Lifileucel and pembrolizumab demonstrated durable antitumor activity and deep response characteristics in ICIs-naive populations (185). Clinical studies in metastatic gastrointestinal tumors verified long-term benefits, with selected TILs plus pembrolizumab showing response durations from 4 months to 3.5 years, including a case of over 10-year survival (186). Phase I trials of mesothelin (MSLN)-targeted CAR-T plus PD-1 inhibitors in malignant pleural mesothelioma exhibited favorable safety profiles and clinically meaningful tumor responses in 18 patients (187).

### 4.5 In combination with antibody-drug conjugates

ADCs target and internalize tumor cell surface antigens, releasing cytotoxic payloads for direct tumor cell killing. This process also induces immunogenic cell death (ICD), releasing DAMPs to activate endogenous antitumor immune responses. ICIs enhance T cell cytotoxic activity by relieving tumor-induced immunosuppression, creating synergistic antitumor effects with ADCs (188). Multiple clinical studies have validated the significant potential of this combination strategy across various solid tumors.

The ASCENT-04 trial demonstrated that in previously untreated PD-L1-positive metastatic triple-negative breast cancer (mTNBC) patients, sacituzumab govitecan (Trodelvy) combined with pembrolizumab (Keytruda) significantly improved PFS compared to pembrolizumab plus chemotherapy (189). In previously untreated HER2-expressing locally advanced or metastatic urothelial carcinoma, disitamab vedotin combined with toripalimab showed superior PFS and OS versus conventional chemotherapy, markedly improving clinical outcomes (190). Preliminary results from the OptiTROP-Lung01 study indicated that in driver gene-negative advanced NSCLC, first-line sacituzumab tirumotecan with tagitanlimab outperformed standard-of-care therapies regardless of PD-L1 expression levels or tumor histology, positioning it as a potential new first-line standard (191). The Phase III KEYNOTE-905 clinical trial evaluated the perioperative (neoadjuvant and adjuvant) administration of enfortumab vedotin (EV) in combination with pembrolizumab in patients with muscle-invasive bladder cancer (MIBC) who were ineligible for or declined cisplatin treatment. Results demonstrated that compared with the control group receiving surgery alone, the EV plus pembrolizumab regimen reduced the risk of tumor recurrence, progression or death by a substantial 60%, and the overall risk of all-cause death by 50% (192).

Combination therapy with ADCs and ICIs has achieved remarkable therapeutic breakthroughs in a variety of solid tumors via a synergistic mechanism characterized by “direct tumor cell killing and immune amplification”, thus providing a novel and highly effective strategy for clinical oncology practice. It should be noted, however, that the efficacy of ADC-ICI combination therapy is highly dependent on the expression level of target antigens; tumor antigen heterogeneity may lead to suboptimal treatment responses in a subset of patients, and the risk of toxicological synergy associated with this combination therapy also requires further comprehensive evaluation.

## 5 Precise patient population stratification and predictive model development

Although immunotherapy has revolutionized the therapeutic landscape of cancer, the marked heterogeneity in patient responses and the potential risk of irAEs (193, 194), underscoring the urgent need for precise patient stratification and dynamic prediction of therapeutic efficacy. Current biomarkers approved by the U.S. FDA, including tumor cell PD-L1 expression, microsatellite instability (MSI), mismatch-repair-deficient (dMMR) status, and high tumor mutational burden (TMB) (195), offer valuable guidance yet present substantial limitations in pan-cancer applications. Static biomarkers such as defective tumor antigen presentation (196) and immune-related gene mutation patterns (197) only provide information from a single spatiotemporal dimension (198), failing to capture the dynamic evolution of the TME. To overcome this bottleneck, AI-based multimodal data fusion and multi-omics deconvolution computation are emerging as the core driving force to replace single static biomarkers and achieve high-precision patient stratification, demonstrating outstanding potential for clinical translation.

The development of deep learning and large language models has enabled non-invasive dissection of tumor–immune crosstalk and optimization of clinical decision-making. Their applications in patient stratification are mainly reflected in breakthroughs across the following dimensions:

Lightweight stratification and prediction models based on routine clinical and blood features transform low-cost, easily accessible data into powerful predictive tools. For instance, the LORIS model, using only six baseline and clinical characteristics, accurately predicts responses to ICIs and survival across multiple solid tumors (199). The SCORPIO model achieves robust ICIs efficacy prediction through routine blood tests (e.g., complete blood count, metabolic panel) and clinical characteristics (age, gender, BMI), demonstrating strong performance in both real-world and phase III clinical settings (200). Such models greatly lower the barrier to implementing precise stratification, especially in resource-limited healthcare settings.

Prediction models based on deep feature extraction from computational pathology and spatial biology can directly capture spatial immune infiltration features invisible to the human eye from routine digital pathological images. The Deep-IO model uses deep learning to analyze hematoxylin and eosin (H&E)-stained whole-slide images from patients with advanced NSCLC, effectively predicting therapeutic responses to ICIs. As an auxiliary biomarker to PD-L1, it successfully identifies potential beneficiaries of immunotherapy among patients with low or negative PD-L1 expression, significantly optimizing the benefit–cost balance of ICI treatment (201). HEX accurately predicts the spatial expression of 40 protein biomarkers from standard H&E-stained pathological slides and generates “virtual” spatial proteomic maps, markedly improving the accuracy of prognosis prediction and immunotherapy response prediction in patients with lung cancer (202).

Multimodal fusion AI large models are advancing toward cross-modal information integration. Trained on a dataset containing 21 million cells, paired H&E sections, and multiplex immunofluorescence (mIF) data for 21 proteins, GigaTIME achieves cross-modal conversion of routine H&E pathological slides into spatial proteomic data and constructs a virtual patient cohort with cellular state information from conventional sections. This virtual cohort revealed 1,234 statistically significant associations among proteins, biomarkers, stages, and survival—analyses previously unfeasible due to the high cost, technical complexity, and low throughput of mIF. It opens a new avenue for large-scale modeling of the TME (203). The Multimodal Transformer with Unified Masked Modeling (MUSK) model performs unsupervised learning across unpaired medical images and text (clinical records), achieving area under the curve (AUC) values of 0.768 and 0.762 for immunotherapy response prediction in lung cancer and gastroesophageal cancer, respectively. Its composite feature extraction substantially outperforms single biomarkers such as PD-L1 or MSI (204).

Furthermore, addressing the common “small sample size” challenge in clinical data, the Clinical Transformer model maximizes the use of limited existing data via self-supervised and progressive transfer learning, providing a new approach for patient stratification in rare mutation subtypes (205).

However, current AI-driven immunotherapy prediction models are far from perfect, and significant limitations and inherent contradictions remain for their clinical translation. First, generalization is poor: most models are heavily trained on retrospective cohorts from single institutions, and their predictive accuracy often declines sharply when applied to samples from different medical centers, ethnic groups, or with divergent tissue processing standards. Second, the “black-box” nature of deep learning leads to a lack of transparent decision logic, creating major regulatory and ethical obstacles. The high stratification probabilities it outputs often lack clear biological interpretation, making it difficult for clinicians to evaluate the rationality of its decisions. Finally, existing models mostly focus on baseline prediction and lack real-time tracking and predictive capacity for the dynamic remodeling of the TME during treatment and acquired resistance driven by the “co-evolution” of the immune system and tumors.

Therefore, the future development of computational tumor immunology must evolve toward explainable AI and a dynamic prediction system integrated with spatial multi-omics. Only through validation in large-scale prospective clinical trials and the establishment of standardized regulatory evaluation frameworks can multimodal AI models truly break through barriers and deliver safe, reliable decision support for precision cancer immunotherapy.

## 6 Conclusions

Cancer immunotherapy has revolutionized the therapeutic paradigm of tumors by activating or remodeling anti-tumor immune responses. It has demonstrated remarkable efficacy in the clinical treatment of a wide spectrum of solid and hematologic malignancies and has become a core research direction in the field of oncology. However, the development of immunotherapy is restricted by several core challenges, including insufficient exploration of novel therapeutic targets, inherent defects of conventional therapies, the lack of strategies for remodeling the TME of “cold” tumors, and the imperfection of the precision treatment system. This review elaborates on the countermeasures for the above four core issues from four dimensions: elucidation of novel immune regulatory mechanisms and target development, technological innovation-driven upgrading of therapeutic models, optimization of combination therapy strategies, and construction of precise patient identification models.

In terms of novel target development, breakthroughs have been achieved from four aspects: differentially expressed molecules in cells, metabolic regulatory networks, the TME, and novel immune checkpoints, providing precise intervention directions for overcoming bottlenecks such as low response rates and frequent drug resistance in immunotherapy. For therapeutic model upgrading, four major technologies, including engineered bacteria, OVs, ACT, and tumor vaccines, have been innovatively modified via gene editing and synthetic biology. These approaches intervene in different links of the anti-tumor immune response, disrupt the immunosuppressive microenvironment, and enhance the effector function of T cells. In the field of combination therapy, ICI-centered combinations with chemotherapy, radiotherapy, OVs, ACT, and ADCs have been verified to exert synergistic anti-tumor effects. Among them, spatiotemporal sequencing, dose optimization, and precise drug compatibility have become the key to improving combined therapeutic efficacy. For precise stratification, multimodal prediction models based on multi-omics and AI have broken through the limitations of traditional static biomarkers, enabling efficient prediction of immunotherapy responses and adverse events, and providing important support for individualized treatment decision-making.

Despite numerous milestone advances in cancer immunotherapy, many critical issues remain to be addressed in this field. Most novel targets are still in the preclinical or early clinical stage, and their effectiveness requires further validation in real-world settings. Fundamental problems have not been completely solved, including the insufficient targeting stability and safety of engineered bacteria, poor systemic delivery efficiency and unsatisfactory monotherapeutic efficacy of OVs, off-target toxicity of ACT and difficulties in solid tumor infiltration, and low efficiency of neoantigen prediction for tumor vaccines. No universal pan-cancer consensus has been reached regarding the optimal sequence, dose window, and compatibility regimens of combination therapy, and personalized combination strategies for different tumor types still warrant in-depth exploration. AI prediction models are confronted with shortcomings such as weak generalization ability, opaque decision-making logic caused by the “black-box” property, and the lack of real-time tracking capability for tumor-immune co-evolution, resulting in an immature clinical translation and regulatory system.

In the future, the development of cancer immunotherapy will heavily rely on interdisciplinary integration and steadily advance toward directions with clearer mechanisms, more efficient technologies, more precise combinations, and more individualized treatment. For novel target development, it is necessary to further integrate multi-omics, single-cell sequencing, spatial biology, and other technologies to deepen the dissection of the tumor-immune interaction network, with a focus on developing dual-functional targets that possess both tumor specificity and immune activation effects. For therapeutic technology innovation, the in-depth application of synthetic biology and nanotechnology will serve as the core driving force. Precise modification of engineered bacteria and OVs will balance targeting and safety; gene editing and cell engineering will optimize the tumor infiltration capacity and persistence of ACT; and AI algorithms will improve the accuracy of neoantigen prediction and the efficiency of delivery systems for tumor vaccines, promoting the transformation of various therapies from preclinical research to large-scale clinical application. For combination therapy strategies, large-sample, multi-center prospective clinical trials should be conducted based on in-depth mechanistic dissection to define the optimal combination regimens, administration sequences, and dose strategies for different tumor types and patient subgroups. Meanwhile, innovative multi-therapy combination models should be explored to systematically reshape the immunosuppressive TME and maximize synergistic anti-tumor effects. For the construction of a precise treatment system, explainable AI will become a core developmental direction. By integrating spatial multi-omics and dynamic clinical monitoring data, dynamic prediction models capable of real-time tracking of TME evolution and immunotherapy responses will be constructed. Furthermore, large-scale prospective clinical trials will be performed to validate model efficacy, and standardized frameworks for model development, regulatory evaluation will be established to break technical barriers.

With the continuous progress of molecular biology, synthetic biology, artificial intelligence, precision medicine, and other fields, the core bottlenecks of cancer immunotherapy will be gradually overcome. Next-generation cancer immunotherapy strategies will achieve comprehensive improvements in efficacy, safety, and accessibility, bringing long-term survival benefits to more tumor patients and ultimately advancing tumor therapy toward the goal of “cure”.

## Glossary

ICIs: immune checkpoint inhibitors
ACT: adoptive cell therapy
OVs: oncolytic viruses
ORRs: objective response rates
PD-1: programmed cell death protein 1
NSCLC: non-small cell lung cancer
IrAEs: immune-related adverse events
TME: tumor microenvironment
scRNA-seq: single-cell RNA sequencing
CIDE: Cancer Immunology Data Engine
AOAH: acyloxyacyl hydrolase
DHODH: dihydroorotate dehydrogenase
MHC: major histocompatibility complex
VDAC2: voltage-dependent anion channel 2
IFN-γ: interferon-γ
PKM2: pyruvate kinase M2
Treg: regulatory T cell
CARs: chimeric antigen receptors
TAMs: tumor-associated macrophages
MDSCs: myeloid-derived suppressor cells
iTregs: induced Tregs
TH1: T helper 1
HCC: hepatocellular carcinoma
CAF: cancer-associated fibroblast
TiBA-1: Tumor-induced B cell abnormality type 1
TiBA-2: Tumor-induced B cell abnormality type 2
TNBC: triple-negative breast cancer
GC: germinal center
EF: extra-follicular
PD-L1: programmed cell death-ligand 1
PF4: platelet factor 4
Arg1: arginase I
CTLA-4: cytotoxic T-lymphocyte-associated protein 4
OS: overall survival
LAG3: lymphocyte activation gene 3
IND: Investigational New Drug
FDA: Food and Drug Administration
Gal-8: galectin-8
OX40L: OX40 ligand
LMs: liposomes
M-CSF: macrophage colony-stimulating factor
NIR: near-infrared
NETMAP: near-infrared light-mediated PadC-based photoswitch
MWA: microwave ablation
NDV-GT: Newcastle disease virus with porcine α1,3GT gene
DCR: disease control rate
ADVs: adenoviruses
NE: neutrophil elastase
PPE: porcine pancreatic elastase
GBM: glioblastoma
TNF-α: tumor necrosis factor-alpha
TCR-T: T cell receptor-engineered T cells
TIL: Tumor-infiltrating lymphocyte
ScFv: single-chain variable fragment
mPFS: median progression-free survival
mOS: median overall survival
UCAR-T: universal CAR-T
HLA: human leukocyte antigen
TAAs: tumor-associated antigens
TSAs: tumor-specific antigens
RFS: recurrence-free survival
HFRT: hypofractionated radiotherapy
LDRT: low-dose radiotherapy
CR: complete responder
MSLN: mesothelin
ICD: immunogenic cell death
dMMR: mismatch-repair-deficient
TMB: tumor mutational burden
AUC: area under the curve
DCs: dendritic cells
PAMPs: pathogen-associated molecular patterns
DAMPs: damage-associated molecular patterns
APCs: antigen-presenting cells.
